# Reviewed article: Rocha VF, Barbosa MS, Neves E, Santos VJ, Rego RFS, Pessoa TL, Ribeiro MT, Santos JLA, Pereira FM, Reis JN. Vancomycin-resistant Enterococci infections in a hospital in Salvador, Bahia, Brazil: a descriptive study, 2021-2023. Epidemiol Serv Saude. 2025:34;20240135

**DOI:** 10.1590/S2237-96222025v34e20240135.b

**Published:** 2025-04-28

**Authors:** Luciene Conterno

**Affiliations:** Universidade de Campinas

**Table:** 

Rating	Excellent	Good	Average	Below average	Poor
Interest		✓			
Quality			✓		
Originality			✓		
Overall	✓				

**Table:** 

Questionnaire	Yes	No	Not applicable
Does the manuscript contain new and significant information to justify publication?	✓		
Does the Abstract (Summary) clearly and accurately describe the content of the article?	✓		
Is the problem significant and concisely stated?	✓		
Are the methods described comprehensively?	✓		
Are the interpretations and conclusions justified by the results?	✓		
Is adequate reference made to other work in the field?	✓		

## Manuscript Structure:

Length of article is: Adequate

Number of tables is: Adequate

Number of figures is: Adequate

Please state any conflict(s) of interest that you have in relation to the review of this paper (state “none” if this is not applicable): None

## Comments

This study highlights critical data regarding hospital outbreaks due to inadequate handling and storage of contaminated materials. This issue likely occurs in numerous Brazilian hospitals.

The study, however, is not original and exhibits some limitations in investigating potential sources of Enterococcus dissemination and characterizing the strains. The advent of the COVID-19 pandemic significantly disrupted health services and the control of nosocomial infections.

One pertinent aspect that could have been discussed is the necessity imposed by the pandemic context to allocate inadequately trained personnel to assist patients in the ICU, potentially contributing to technical failures during patient care.

The authors could have conducted an epidemiological assessment, such as a case-control study, to characterize temporal relationships and identify risk factors cautiously.

Despite these limitations, it is crucial to recognize that these outbreaks occurred and that, from an operational standpoint, they were effectively controlled. 

